# BAP1 Loss Promotes Suppressive Tumor Immune Microenvironment via Upregulation of PROS1 in Class 2 Uveal Melanomas

**DOI:** 10.3390/cancers14153678

**Published:** 2022-07-28

**Authors:** Christopher J. Kaler, James J. Dollar, Anthony M. Cruz, Jeffim N. Kuznetsoff, Margaret I. Sanchez, Christina L. Decatur, Jonathan D. Licht, Keiran S. M. Smalley, Zelia M. Correa, Stefan Kurtenbach, J. William Harbour

**Affiliations:** 1 Bascom Palmer Eye Institute, Sylvester Comprehensive Cancer Center and Interdisciplinary Stem Cell Institute, University of Miami Miller School of Medicine, Miami, FL 33136, USA; cjk126@med.miami.edu (C.J.K.); jjd175@med.miami.edu (J.J.D.); amc606@med.miami.edu (A.M.C.); jkuznetsov@med.miami.edu (J.N.K.); msanchez4@med.miami.edu (M.I.S.); cdecatur@med.miami.edu (C.L.D.); zcorrea@med.miami.edu (Z.M.C.); stefan.kurtenbach@med.miami.edu (S.K.); 2 University of Florida Health Cancer Center, University of Florida Cancer and Genetics Research Complex, Gainesville, FL 32610, USA; jdlicht@ufl.edu; 3 Department of Tumor Biology, Moffitt Cancer Center & Research Institute, Tampa, FL 33612, USA; keiran.smalley@moffitt.org; 4 Department of Ophthalmology and Harold C. Simmons Comprehensive Cancer Center, University of Texas Southwestern Medical Center, Dallas, TX 75390, USA

**Keywords:** uveal melanoma, BAP1, PROS1, MERTK, macrophage, tumor immune microenvironment, metastasis

## Abstract

**Simple Summary:**

Uveal melanoma is a highly metastatic cancer of the eye which is notoriously resistant to therapy. Elucidating the mechanisms of metastasis in order to devise effective therapies has been a major challenge. The strongest genetic risk factor for metastasis in uveal melanoma is the mutational inactivation of the *BAP1* tumor-suppressor gene. However, it remains unknown how BAP1 loss promotes tumor progression. Here, we show that BAP1 loss leads to increased expression of PROS1 in uveal melanocytes and melanoma cells, which in turn leads to phosphorylation and activation of the receptor tyrosine kinase MERTK on adjacent macrophages, driving them into a suppressive M2-polarized state. This mechanism could help explain the suppressive tumor immune microenvironment that is characteristic of BAP1-mutant uveal melanomas, and it suggests that BAP1 loss may lead to metastasis at least in part by facilitating immune escape. These findings provide new insights into the role of BAP1 in uveal melanoma, and they nominate new strategies for increasing the efficacy of immunotherapy in this cancer.

**Abstract:**

Uveal melanoma (UM) is the most common primary cancer of the eye and is associated with a high rate of metastatic death. UM can be stratified into two main classes based on metastatic risk, with class 1 UM having a low metastatic risk and class 2 UM having a high metastatic risk. Class 2 UM have a distinctive genomic, transcriptomic, histopathologic, and clinical phenotype characterized by biallelic inactivation of the *BAP1* tumor-suppressor gene, an immune-suppressive microenvironment enriched for M2-polarized macrophages, and poor response to checkpoint-inhibitor immunotherapy. To identify potential mechanistic links between BAP1 loss and immune suppression in class 2 UM, we performed an integrated analysis of UM samples, as well as genetically engineered UM cell lines and uveal melanocytes (UMC). Using RNA sequencing (RNA-seq), we found that the most highly upregulated gene associated with BAP1 loss across these datasets was *PROS1*, which encodes a ligand that triggers phosphorylation and activation of the immunosuppressive macrophage receptor MERTK. The inverse association between *BAP1* and *PROS1* in class 2 UM was confirmed by single-cell RNA-seq, which also revealed that *MERTK* was upregulated in CD163+ macrophages in class 2 UM. Using ChIP-seq, BAP1 knockdown in UM cells resulted in an accumulation of H3K27ac at the *PROS1* locus, suggesting epigenetic regulation of *PROS1* by BAP1. Phosphorylation of MERTK in RAW 264.7 monocyte–macrophage cells was increased upon coculture with BAP1^−/−^ UMCs, and this phosphorylation was blocked by depletion of PROS1 in the UMCs. These findings were corroborated by multicolor immunohistochemistry, where class 2/BAP1-mutant UMs demonstrated increased PROS1 expression in tumor cells and increased MERTK phosphorylation in CD163+ macrophages compared with class 1/BAP1-wildtype UMs. Taken together, these findings provide a mechanistic link between BAP1 loss and the suppression of the tumor immune microenvironment in class 2 UMs, and they implicate the PROS1–MERTK pathway as a potential target for immunotherapy in UM.

## 1. Introduction

Uveal melanoma (UM) is the most common primary cancer of the eye and is often associated with fatal metastasis [1]. UM can be stratified into two prognostically significant subtypes, with class 1 UM having a low metastatic risk and class 2 UM having a high metastatic risk [2,3]. Biallelic inactivation of the tumor-suppressor *BAP1* is the distinctive genomic feature of class 2 UM, which occurs through the mutation of one allele and the whole-chromosome loss of the other allele [4]. While we and others have extensively investigated the downstream effects of BAP1 loss in UM cells [5,6,7], the mechanism by which BAP1 loss leads to metastasis remains unclear.

Despite the increasingly effective management of primary UM, there has been no corresponding improvement in patient survival [8], due to a propensity for early micrometastasis and immune escape [9]. UM is an immunoresistant tumor type that responds poorly to checkpoint-inhibitor immunotherapy [10]. This is due, at least in part, to a suppressive tumor immune microenvironment (TIME), including an enrichment of M2 polarized (M2) macrophages, T cells expressing checkpoint or “exhaustion” markers, and increased HLA expression on tumor cells [11,12,13,14,15]. Importantly, the suppressive TIME in UM is strongly associated with mutational inactivation of *BAP1* [11,12,16], suggesting a mechanistic link between genomic aberrations and immune suppression.

While it is now widely recognized that the TIME plays a critical role in cancer progression and therapeutic resistance [17], how the tumor genome shapes the TIME remains poorly understood. Here, we used an integrative approach to explore the mechanistic relationship between *BAP1* mutations and the TIME in UM.

## 2. Materials and Methods

### 2.1. Cell Lines

UMC026 cells with and without CRISPR-Cas9-mediated deletion of the first exon of BAP1 (*BAP1*^−/−^) were established and cultured in our laboratory from normal uveal melanocytes in a patient undergoing enucleation, as previously described [18]. RAW 264.7 monocyte–macrophage-like cells (ATCC, Manassas, Virginia) were grown in DMEM media with 10% Tet-Free FBS at 37 °C in 20% oxygen and 5% CO_2_. MP41 and MP46 cells were gifts from Dr. Sergio Roman-Roman [19] and were cultured as previously described [20]. Mel202 and 92.1 were gifts from Drs. B. Ksander and M. Jager, respectively. UMC026, 92.1, Mel202, and MP41 were engineered to allow tetracycline-inducible knockdown of BAP1 as previously described [21]. The BAP1-mutant cell line MP46 UM was engineered to allow for tet-inducible expression of exogenous BAP1 by stable lentiviral integration of pLV-TET-HA-BAP1-WT and pLVX-TET-ON (Clontech) constructs. pLV-TET-HA-BAP1-WT was created by PCR amplification and subsequent recombination of a full-length BAP1 cDNA fragment into pLV-TET-PURO (Addgene #26430). The plasmids were packaged into lentiviral particles by transient co-transfection into HEK293T cells with pMD2G and psPAX2 packaging plasmids using JetPrime reagent (Polyplus), and the virally transduced MP46 cells selected with puromycin (2 µg/mL) and geneticin (500 µg/mL).

### 2.2. RNA Sequencing (RNA-Seq)

RNA-seq was performed as previously described [21]. Briefly, RNA was isolated with Direct-zol RNA kit (Zymo), and melanin pigment was removed using OneStep PCR Inhibitor Removal Kit (Zymo), according to the manufacturers’ instructions. Libraries were prepared and sequencing was performed by the Sylvester Comprehensive Cancer Center Oncogenomics Shared Resource at the University of Miami. Sequencing quality was assessed using FastQC (v0.11.3, Simon Andrews, Cambridge, UK). Reads were trimmed using Trim Galore (v0.6.5, Felix Krueger, Cambridge, UK) [22], aligned to the human genome builder hg38/GRCH38 using STAR (v2.5, Alexander Dobin, CA, USA) [23] and counts were generated using RSEM (v1.3.3, Bo Li and Colin Dewey, Boston, MA, USA) [24]. Venn diagram was generated by overlap of RNA-seq datasets included genes with >30% upregulation, and FPKM > 10. Significance of PROS1 expression in shBAP1 and BAP1 tumor samples was calculated with a ratio-paired *t*-test of normalized read counts across all datasets.

### 2.3. Immunoblotting

Immunoblotting was performed as previously described [20]. Briefly, cells were harvested from culture dishes, suspended in RIPA buffer with protease and phosphatase inhibitors on ice, sonicated at 50% power for 10 s on ice, and protein-quantified by BCA assay. Equivalent cell protein quantities were mixed with 1% SDS, heated for 10 min at 75 °C, and electrophoresed through TGX polyacrylamide gels (Bio-Rad, Hercules, CA, USA), or frozen at −20 °C. Primary antibodies included anti-BAP1 (Santa Cruz Biotechnologies, Dallas, TX, USA, sc-28383), anti-PROS1 (Catalogue #16910-1-AP, Proteintech, Rosemont, IL, USA), or anti-β-actin (Catalogue #66009-1-IG, Proteintech, Rosemont, IL, USA) antibodies and HRP secondary antibodies (Catalogue #7076S and 7074P2, Cell Signaling Technology, Danvers, MA, USA). Blot imaging was performed with the Bio-Rad ChemiDoc XRS Imaging System (Bio-Rad, Hercules, CA, USA) and densitometric signal quantitation was performed using ImageJ (https://imagej.nih.gov/ij/, accessed on 22 November 2021).

### 2.4. ChIP-Seq

Chromatin immunoprecipitation (ChIP) followed by next-generation sequencing (ChIP-seq) was performed using 20 million cells per experiment, which were crosslinked for 7 min with 1% formaldehyde. Chromatin was sonicated to an average fragment size of 200–500 base pairs with a Covaris M220 sonicator. A measure of 10 μg of antibody was used for each ChIP experiment. Libraries were prepared using the NEBNext Ultra 2 kit and sequenced by the University of Miami Oncogenomics Shared Resource with >20 million reads per sample. Reads were quality filtered by Trim Galore! [22] and aligned to the hg38 genome with Bowtie2 [25]. Normalized coverage tracks were generated with MACS2 [26], and plotted with SparK [27].

### 2.5. Single-Cell RNA Sequencing (scRNA-Seq)

A total of 59,915 cells from 11 UM samples were analyzed by scRNA-seq as previously described [11]. Using the first 20 principal components of variably expressed genes, dimensionality reduction was conducted with Seurat (version 4.1.0, Yuhan Hao, New York, NY, USA) using Uniform Manifold Approximation and Projection (UMAP) methodology [28,29]. Cell type annotations were assigned as previously described [11]. Differentially expressed genes were identified using Wilcoxon Rank Sum test of log normalized counts. Dimensional reduction plots, dot plots, and scatter plots were generated using Seurat. Significance of correlation between the expression of genes was conducted in R using Spearman’s rank-based measure of association.

### 2.6. Multicolor Immunohistochemistry

Multicolor immunohistochemistry was performed using HistoWiz Inc. (Brooklyn, NY, USA) on primary UM samples that were collected after enucleation, fixed in formalin, embedded in paraffin, and placed onto glass slides in 4 μm sections. Sequential tyramide (Akoya OPAL, Akoya Biosciences, Marlborough, MA, USA)-based immunofluorescence was performed on a Bond Rx autostainer (Leica Biosystems, Deer Park, IL, USA) with EDTA pH 9.0 Heat-Induced Epitope Retrieval (HIER) for 40 min. Antibodies used were sequentially included: phospho-MERTK (Cataogue #PMKT-140AP, FabGennix, Frisco, TX, USA) at 1:50 dilution; PROS1 (Catalogue # ab280885, Abcam, Waltham, MA, USA) at 1:175 dilution; CD163 (Histowiz, Brooklyn, NY, USA) at 1:100 dilution; and BAP1 (Catalogue # ab255611, Abcam, Waltham, MA, USA) at 1:100 dilution. Slides were counterstained with DAPI, then cover-slipped with Prolong Diamond Antifade Mounting Medium (Catalogue # P36961, Thermo Fisher Scientific, Waltham, MA, USA) to prevent bleaching and signal loss. Whole-slide scanning (20×) was performed on an Akoya Polaris IF Scanner (Akoya Biosciences, Marlborough, MA, USA) with matching OPAL filters o570, o620, o480 and o520. VisioPharm Artificial Intelligence (AI) image analysis (VisioPharm, Hoersholm, Denmark) was performed on 60 regions of interest measuring 466 µm × 349 µm from class 1 (UMM065, UMM079) and class 2 (UMM063, UMM069) primary UM samples (Supplementary Table S1). A total of 91,532 cells were analyzed and the percentage of cells staining positive for BAP1, PROS1, CD163, and Phospho-MERTK was determined. Additionally, the percentage of cells that were co-stained for CD163 and phospho-MERTK was determined.

### 2.7. ELISA

Media was collected from cell culture plates following 72 h incubation of UMC026 cells with and without BAP1 knockout. Media samples (1 mL) were centrifuged at 1000× *g* for 10 min, and 800 µL was removed and frozen at −80 °C. Cell count was determined for each culture plate by trypsinization of cells (2 mL, 0.5% trypsin) and quantification by spectrophotometer. PROS1 protein quantification was performed using a commercially validated, enzyme-linked immunosorbent assay (Catalogue # MBS9427967, MyBioSource, Inc., San Diego, CA, USA) for three different sets of media from UMC026 cells with and without BAP1 knockout. ELISA measurements were performed in triplicate using a VersaDoc spectrophotometer instrument (Bio-Rad; Hercules, CA, USA).

### 2.8. Confocal Immunocytochemistry

UMC026 cells with and without BAP1 knockout were grown in culture on glass coverslips coated with poly-l-lysine. Cells were fixed in 4% paraformaldehyde (*v*/*v* in PBS) for 10 min, washed 2× in PBS and incubated in 0.8% glycine (*v*/*v* in PBS) for 10 min. Cells were then permeabilized for 30 min in 0.05% Tween-20 (*v*/*v* in PBS), incubated in 0.27% ammonium chloride (*v*/*v* in PBS) for 10 min, and washed 3× in PBS. Cells were blocked with 5% BSA, 1% NGS and 0.5% Tween-20 (*v*/*v* in TBS) for 1 h and washed 3× in TBS. Cells were incubated in anti-PROS1 primary antibody (Proteintech, Rosemont, Illinois, 16910-1-AP) overnight at 4 °C, washed 3× in TBS and then incubated in secondary antibody (Cell Signaling Technology, Danvers, MA, USA, 7074P2) for 1 h at room temperature. Samples were then washed 3× in TBS, mounted with SlowFade Diamond Antifade mounting medium with DAPI (Thermo Fisher Scientific, Waltham, MA, USA), and imaged using an SP8 Leica laser scanning confocal microscope (Leica Microsystems Inc, Buffalo Grove, IL, USA).

### 2.9. Coculture Experiments

MERTK activation in RAW 264.7 cells was assessed by immunoprecipitation and immunoblot as previously described [30] following 12 h coculture with BAP1^+/+^ or BAP1^−/−^ UMC026 cells, or BAP1^−/−^ UMC026 cells treated with siRNA against PROS1 or with nontargeting control siRNA using commercially validated siRNA oligonucleotides and lipofectamine RNAi max reagents (Thermo Fisher Scientific, Carlsbad, CA, USA). Coculture was initiated 48–72 h after treatment with siRNA. UMC026 cells (2.5 × 10^6^) were cultured alone or with RAW 264.7 cells (2.5 × 10^6^; plated 24 h prior) in DMEM media with 10% Tet-Free FBS at 37C in 20% oxygen and 5% CO_2_ for 12 h. Cocultures were treated with 120 μM pervanadate (prepared fresh by combining 20 mM sodium orthovanadate in 0.9× PBS in a 1:1 ratio with 0.3% hydrogen peroxide in PBS for 15–20 min at room temperature) for 3 min prior to collection. Cell lysates were prepared in 50 mM HEPES (pH 7.5), 150 mM NaCl, 10 mM EDTA, 10% glycerol, and 1% Triton X-100, supplemented with protease inhibitors (Sigma-Aldrich, St. Louis, MO, USA). MERTK was immunoprecipitated with anti-Mer (R&D Systems, Minneapolis, MN, USA) and Protein G beads. Phospho-MERTK was detected by immunoblotting using an anti-phospho-Mer (FabGennix, Frisco, TX, USA) antibody. Nitrocellulose membranes were then stripped, and total-MERTK detected using anti-Mer antibody (R&D Systems, Minneapolis, MN, USA). The coculture experiments were performed in triplicate and the ratios of phosphorylated- to total-MERTK protein levels were obtained by densitometry using ImageJ.

### 2.10. Statistical Analysis

A two-tailed *t*-test was used for continuous data and chi square analysis for categorical data, using GraphPad Prism software version 8.0 for Windows, GraphPad Software (San Diego, CA, USA, www.graphpad.com, accessed on 15 March 2022). For scRNA-seq differential expression analysis, two-sided nonparametric Wilcoxon rank sum test with Bonferroni correction using all genes was used. Pearson correlation was used for correlating expression between genes.

### 2.11. Accession Codes

Submission of the RNA-Seq and ChIP-seq data generated in this study to the Gene Expression Omnibus is in process.

## 3. Results

### 3.1. BAP1 Regulates PROS1 by Epigenetic Mechanisms

**Figure 1:**
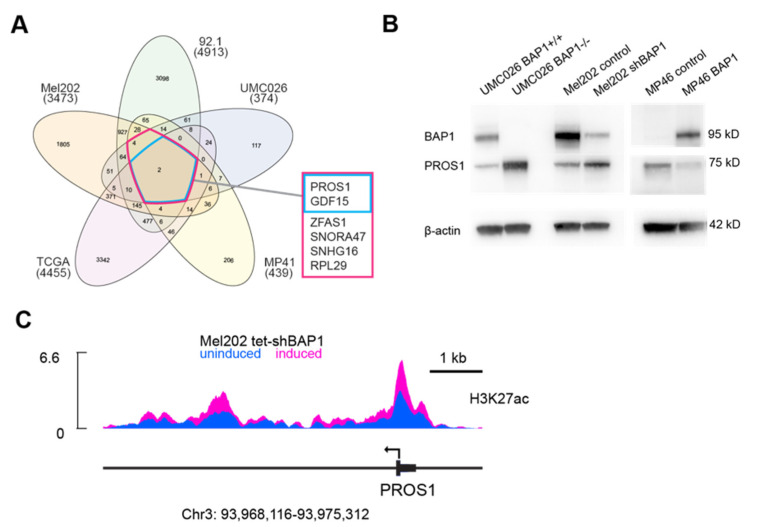
PROS1 expression is regulated by BAP1 in uveal melanocytes and uveal melanoma cells. (**A**) Summary of RNA sequencing data from BAP1-wildtype Mel202, 92.1, and MP41 uveal melanoma cells, and UMC026 uveal melanocytes with or without shRNA-mediated knockdown of BAP1, and 80 UM samples from The Cancer Genome Atlas (TCGA) data repository with or without mutational inactivation of BAP1. Numbers in parenthesis indicate the number of genes upregulated by loss of BAP1. Numbers within the Venn diagram indicate the number of overlapping genes between indicated subsets of genes. PROS1 was one of only two genes that was upregulated across all datasets. (**B**) Immunoblots confirming upregulation of PROS1 protein following knockout of BAP1 in UMC026 cells, or knockdown of BAP1 in Mel202 cells. Conversely, ectopic expression of BAP1 in MP46 BAP1-mutant class 2 uveal melanoma cells resulted in downregulation of PROS1. PROS1/β-actin densitometry ratios (lanes 1–6): 0.29592, 1.2059, 0.61066, 1.1644, 0.34334, 0.10613 (**C**) ChIP-seq analysis of H3K27ac at the PROS1 locus in Mel202 cells with or without doxycycline-induced, shRNA-mediated knockdown of BAP1.

To identify the genes regulated by BAP1, we analyzed RNA-seq data for differentially expressed genes in three UM cell lines (92.1, Mel202, MP41) and one cell line derived from normal human uveal melanocytes (UMC026) following shRNA knockdown of BAP1, and in 80 human UMs from The Cancer Genome Atlas (TCGA) database that were wildtype versus mutant for BAP1. By plotting genes with 30% increase in FPKM and a minimum of 10 FPKM, we found that only two genes were upregulated across all of these datasets, *PROS1* and *GDF15* (Figure 1A), both of which have been linked to immunosuppressive macrophage polarization [31,32]. Upregulation was significant for both *PROS1* (*p* = 0.0027) and *GDF15* (*p* = 0.0194) when comparing BAP1-competent samples with BAP1-deficient samples across all datasets. Here, we focused on *PROS1* and its potential role in suppressing the TIME in BAP1-deficient UM. Deletion of BAP1 in UMC026 cells and knockdown of BAP1 in Mel202 (class 1) cells resulted in upregulation of PROS1, whereas ectopic expression of BAP1 in BAP1-deficient MP46 (class 2) cells resulted in downregulation of PROS1 (Figure 1B and Supplementary Figures S1 and S2). Furthermore, knockdown of BAP1 resulted in increased acetylation of histone H3 at lysine 27 (H3K27ac) around the *PROS1* locus (Figure 1C). These findings suggest that BAP1 represses *PROS1* at least in part by epigenetic mechanisms, and that BAP1 loss leads to increased *PROS1* expression.

### 3.2. Single-Cell Sequencing Analysis of PROS1 and MERTK in Uveal Melanomas

**Figure 2:**
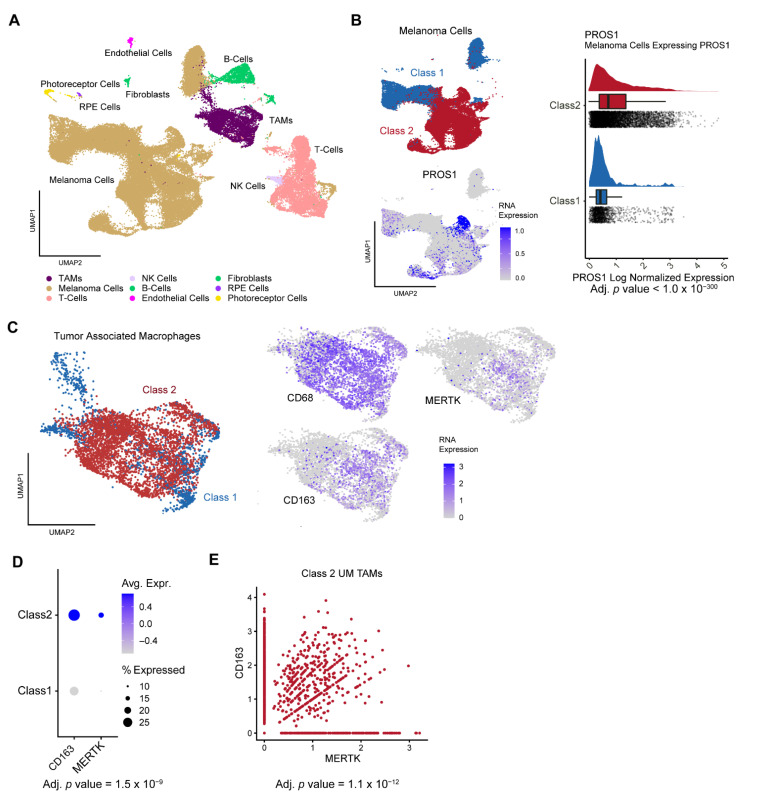
Single-cell RNA sequencing analysis of *PROS1* and *MERTK* in uveal melanomas. (**A**) UMAP dimensionality reduction plot of 59,915 neoplastic and non-neoplastic cells from 11 uveal melanoma samples, with cell types indicated in the legend. (**B**) Left: UMAP plot of the 42,230 uveal melanoma cells in the dataset, with colors indicating gene expression profile (GEP) class 1 (*blue*) and class 2 (*red*). Right: corresponding UMAP plot demonstrating log normalized expression of *PROS1*, as indicated by heatmap. (**C**) Left: UMAP plot of the 5053 monocytes/macrophages in the dataset, with colors indicating GEP class 1 (*blue*) and class 2 (*red*) of the tumors from which the cells were derived. Right: corresponding UMAP plots demonstrating log normalized expression of *CD68*, *CD163,* and *MERTK*, as indicated by a heatmap. (**D**) Dot plot demonstrating *MERTK* expression in monocytes/macrophages with respect to GEP class status of the tumors from which the cells were derived. (**E**) Scatter plot demonstrating association between expression of MERTK and CD163 in monocytes/macrophages from class 2 tumors.

To further explore the relationship between BAP1 and PROS1, we analyzed single-cell RNA sequencing from 11 human UM samples, as previously described [11] (Figure 2A). There was a strong association between BAP1-mutant class 2 tumors and PROS1 expression (adj. *p*-value < 10^−300^) (Figure 2B). We then performed a further analysis limited to tumor-associated macrophages, which clustered according to GEP class (Figure 2C). Macrophages derived from class 2 tumors demonstrated significantly increased expression of the macrophage receptor tyrosine kinase *MERTK* (adj. *p*-value = 1.5 × 10^−9^) (Figure 2D), which was strongly associated with expression of the M2 polarization marker CD163 (adj. *p*-value 1.1 × 10^−12^) (Figure 2E).

### 3.3. Validation of PROS1 Upregulation and MERTK Activation in Class 2 Uveal Melanomas

**Figure 3:**
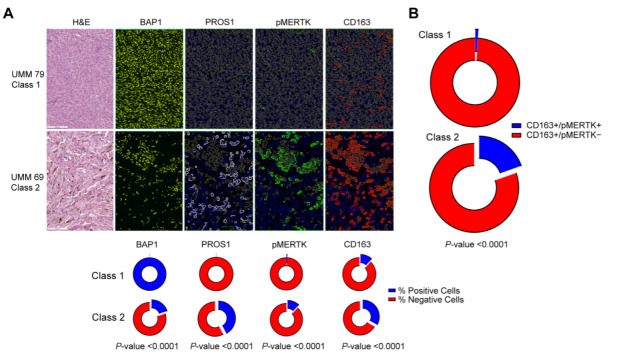
Multicolor immunohistochemistry of PROS1, MERTK, and CD163 in uveal melanomas. (**A**) Top, representative photomicrographs of class 1 and class 2 uveal melanomas analyzed with hematoxylin and eosin (H&E) and with immunostaining for BAP1, PROS1, phosphorylated MERTK (phospho-MERTK), and CD163. Scale bar (lower left), 100 µm. Bottom: donut plots summarizing percentage of cells that were positive for each biomarker with respect to GEP tumor class 1 versus class 2. (**B**) Donut plot demonstrating the proportion of CD163+/phospho-MERTK+ cells with respect to GEP tumor class 1 versus class 2. Total number of cells analyzed and number (and percentage) of cells staining positive for each indicated protein are summarized in Table 1.

Consistent with these findings, multicolor immunohistochemistry in human class1/BAP1-wildtype and class2/BAP1-mutant UM samples revealed a significant association between BAP1 loss and PROS1 upregulation in UM cells and MERTK phosphorylation in tumor-associated macrophages (Table 1, Figure 3A and Supplementary Figures S3–S6). Furthermore, macrophages expressing the M2 polarization marker CD163 were enriched in BAP1-mutant UM samples as previously reported [33]. In BAP1-mutant UM samples, the subset of CD163-positive cells also positive for phospho-MERTK (double-positive cells) was significantly higher than in class 1 UM cells (Figure 3B), consistent with increased MERTK signaling in immunosuppressive macrophages of BAP1-mutant vs. BAP1-wildtype UM.

### 3.4. PROS1 Upregulation following BAP1 Loss Triggers MERTK Phosphorylation in Macrophages

**Figure 4:**
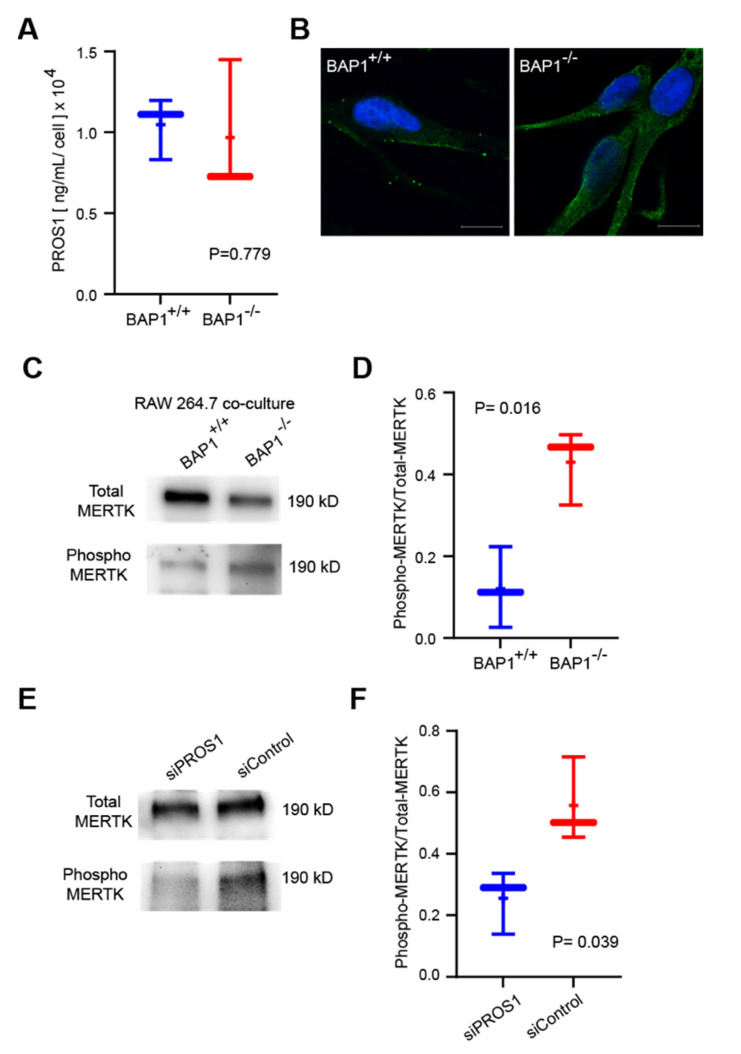
Loss of BAP1 in uveal melanocytes activates MERTK in macrophages in a PROS1-dependent manner. (**A**) Soluble PROS1 protein levels measured by ELISA in media from cultured UMC026 cells with or without knockout of BAP1. (**B**) Confocal microscopy of UMC026 cells with or without knockout of BAP1 immunostained for PROS1. Scale bar (lower right), 10 µm. (**C**) Representative immunoblot of lysates from RAW 264.7 cells cocultured with UMC026 cells with or without knockout of BAP1, immunoprecipitated with total-MERTK antibody, and probed with either total-MERTK or phospho-MERTK antibody. Immunoprecipitated MERTK is undetectable when UMC026 cells are cultured independently of RAW 264.7 cells (Supplementary Figure S11). Phospho-MERTK/total-MERTK densitometry ratios (lanes 1–2): 0.11181, 0.46660. (**D**) Densitometric analysis of immunoblots from triplicate coculture experiments represented in panel C. (**E**) Representative immunoblot of lysates from RAW 264.7 cells cocultured with UMC026 cells knocked out for BAP1, with or without siRNA-mediated knockdown of PROS1, immunoprecipitated with total-MERTK antibody, and probed with phospho-MERTK antibody. Phospho-MERTK/total-MERTK densitometry ratios (lanes 1–2): 0.33620, 0.71485. (**F**) Densitometric analysis of immunoblots from triplicate coculture experiments represented in panel E.

PROS1 can function as a secreted protein, and it can also be localized to the cell membrane and cytoplasm [34,35,36,37]. In UMC026 cells, knockout of BAP1 did not result in an increase in secreted PROS1 in the cell culture media (Figure 4A, Supplementary Figure S7), whereas we found PROS1 localized to the cell membrane and cytoplasm (Figure 4B and Supplementary Figure S8), suggesting that it may function through cell–cell interaction rather than paracrine signaling in this setting. PROS1 is known to promote M2 macrophage polarization by stimulating phosphorylation of the MERTK receptor [31,37,38,39]. Thus, we performed coculture experiments to study the effect of BAP1 loss in UMC026 cells on MERTK phosphorylation in RAW 264.7 monocyte–macrophage cells [40,41]. Indeed, BAP1 knockout in UMC026 cells resulted in a significant increase in MERTK phosphorylation on cocultured RAW 264.7 cells (Figure 4C,D and Supplementary Figure S9), which was blocked by knockdown of PROS1 in BAP1-KO UM026 cells (Figure 4E,F and Supplementary Figures S9 and S10).

## 4. Discussion

Inactivation of BAP1 is the single most consistent mutation associated with metastatic death in UM [4,42], and yet it remains unclear how BAP1 loss promotes metastasis. BAP1 is a deubiquitinating enzyme with many binding partners and substrates, and it has been proposed to affect a wide range of processes such as transcriptional regulation, DNA repair, and metabolism [43]. Interestingly, loss of BAP1 in uveal melanocytes and UM cells results only in a subtle phenotype in vitro [7], suggesting that the dramatic metastatic phenotype associated with BAP1 loss in vivo may be the result of complex multicellular interactions that are not adequately captured by cell culture experiments. BAP1-mutant UMs display a suppressive TIME enriched for M2 polarized macrophages and T cells expressing checkpoint or “exhaustion” markers such as LAG3, TIM3, and TIGIT [11,12,16,44], similar to findings in other cancer types [45]. Consequently, BAP1 loss may promote metastasis at least in part by allowing tumor cells to evade the patient’s immune response. However, it remains unknown how BAP1 mutations may mechanistically lead to immune suppression.

As a potential explanation, we found here that BAP1 loss results in upregulation of PROS1 in UM cells through epigenetic mechanisms involving H3K27ac accumulation at the *PROS1* locus, consistent with our previous findings [18]. Upregulation of PROS1 has also been associated with increased metastatic risk in other cancer types [46,47,48]. PROS1 is a ligand and agonist of the MERTK receptor [49], which when activated by phosphorylation triggers signaling pathways in macrophages that suppress proinflammatory M1 polarization and promote anti-inflammatory M2 polarization [37,39,50]. M2 polarized macrophages lead to further suppression of the TIME by secreting cytokines that inhibit T cells and other immune cell types [38]. Indeed, M2 macrophages may be a primary driver of the suppressive TIME in UM [13], and they are associated with suppression of T cells related to therapeutic resistance to tebentafusp, a T-cell redirection therapy, and the only FDA-approved medication for metastatic UM [51].

**Figure 5:**
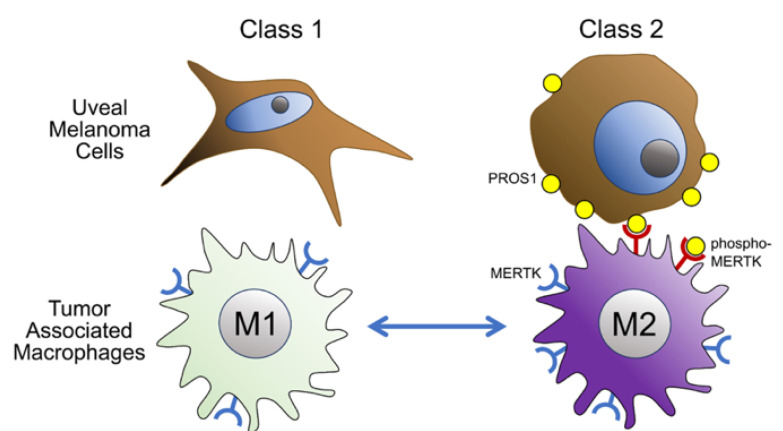
Proposed model for how BAP1 loss leads to suppression of the tumor immune microenvironment. In class 1 BAP1-wildtype uveal melanomas, tumor cells express low levels of PROS1, and tumor-associated macrophages are mostly M1-polarized with low MERTK expression and low MERTK phosphorylation. In BAP1-mutant class 2 uveal melanomas, PROS1 is upregulated since it is no longer repressed by BAP1. Mostly, membrane-bound PROS1 on tumor cells interacts with MERTK on nearby macrophages, leading to phosphorylation of MERTK and activation of downstream signaling that promotes M2 polarization.

The critical role of cancer genomic aberrations in promoting immune evasion, cancer evolution, and cancer metastasis has become increasingly apparent [52,53]. Our findings reveal a potential mechanistic link between BAP1 mutations and immune evasion in UM via transcriptionally mediated increased PROS1 expression and phospho-activation of tumor-associated macrophage MERTK receptors (Figure 5), and they propose new possibilities for overcoming resistance to immunotherapy in UM.

We chose to employ a coculture model in this study because UM cell lines are not more metastatic in existing animal models following BAP1 knockdown [7]. We believe that one reason for this lack of adequate animal model is that current models rely largely on immunodeficient mice, where the mechanism of immunosuppression associated with BAP1 loss that we reveal here would not be operative. It is becoming increasingly clear that many tumor suppressors drive cancer progression by their effects on immune evasion [54].

A strength of our study was the use of UMC026 cells derived from normal human uveal melanocytes instead of uveal melanoma cell lines in the coculture experiments. This allowed us to validate that the findings were specific to BAP1 loss and not the result of private genomic aberrations peculiar to a specific UM cell line [55]. Due to interspecies ligand compatibility for MERTK [45], murine RAW 264.7 cells are preferable to human monocyte/macrophage cell lines for coculture experiments because they do not require a differentiation step before use and manifest low levels of phosphorylated MERTK at baseline [40]. Reagents used for human monocyte/macrophage differentiation such as PMA (Phorbol 12-myristate 13-acetate) and LTA (Lipoteichoic acid) can themselves perturb MERTK expression and signaling, which would mask the phenomenon we sought to evaluate in these experiments [40].

Indeed, the MERTK inhibitor sitravatinib has been shown to circumvent resistance to immune checkpoint blockade in immunoresistant cancer types through its effects on the suppressive TIME [38]. These findings warrant further investigation using in vivo models to explore the potential role for targeting the PROS1–MERTK pathway in UM.

## 5. Conclusions

Mutational inactivation of BAP1 in UM may lead to a suppressive TIME at least in part by upregulation of PROS1 in tumor cells and phospho-activation of MERTK in tumor-associated macrophages. These findings nominate MERTK as a potential target for inhibition to increase the efficacy of immunotherapy in UM.

## Figures and Tables

**Table 1:** Summary of multicolor immunohistochemistry in primary uveal melanomas.

	Number of Cells ^a^	BAP1 ^b^	PROS1	Phospho-MerTK	CD163	CD163 and Phospho-MerTK
Class 1	59,990	59,945 (99.93%)	43 (0.072%)	496 (0.83%)	7098 (11.89%)	77 (1.09%)
Class 2	31,542	6289 (19.94%)	13,301 (42.17%)	3889 (12.27%)	10,476 (33.06%)	2051 (19.58%)
*p*-value	NA	<0.0001	<0.0001	<0.0001	<0.0001	<0.0001

^a^ Total number of cells analyzed from two class 1 and two class 2 primary uveal melanomas. ^b^ Number (and percentage) of cells staining positive for each indicated protein.

## Data Availability

Submission of the RNA-Seq and ChIP-seq data generated in this study to the Gene Expression Omnibus is in process.

## Supplementary Materials

The following supporting information can be downloaded at: https://www.mdpi.com/article/10.3390/cancers14153678/s1. Table S1: Primary UM samples demographic data, Figure S1: Densitometric quantitation of multiple blots for UMC026 and Mel202 cells with and without BAP1 knockout/knockdown, Figure S2: Full Western blot images for Figure 1, Figures S3–S6: Multicolor immunohistochemistry for 60 regions from class 1 (UMM065, UMM079) and class 2 (UMM063, UMM069) uveal melanoma, Figure S7: ELISA standard curve documenting linearity of analyte concentration, Figure S8: Confocal microscopy of UMC026 cells with or without BAP1 knockout immunostained for PROS1, Figure S9: Full Western blot images for Figure 4, Figure S10: Confirmation of PROS1 knockdown in UMC026 BAP1-negative cells used in RAW 264.7 cell coculture functional assays, Figure S11: Immunoprecipitated MERTK expression was undetected when UMC026 cells were cultured without RAW 264.7 cells.
