# Supplementary material for: BAP1 Loss Promotes Suppressive Tumor Immune Microenvironment via Upregulation of PROS1 in Class 2 Uveal Melanomas
Source: Cancers (Basel). 2022 Jul 28;14(15):3678. doi: 10.3390/cancers14153678 (PMC9367253; doi:10.3390/cancers14153678)
Supplement: Supplementary file 1 [file cancers-14-03678-s001.zip › cancers-1789709-supplementary.pdf]

Table S1. Primary UM samples demographic data.

	Age	Gender	Survival Status
UMM063	66	M	Alive
UMM065	44	F	Alive
UMM069	80	F	Expired
UMM079	81	F	Alive

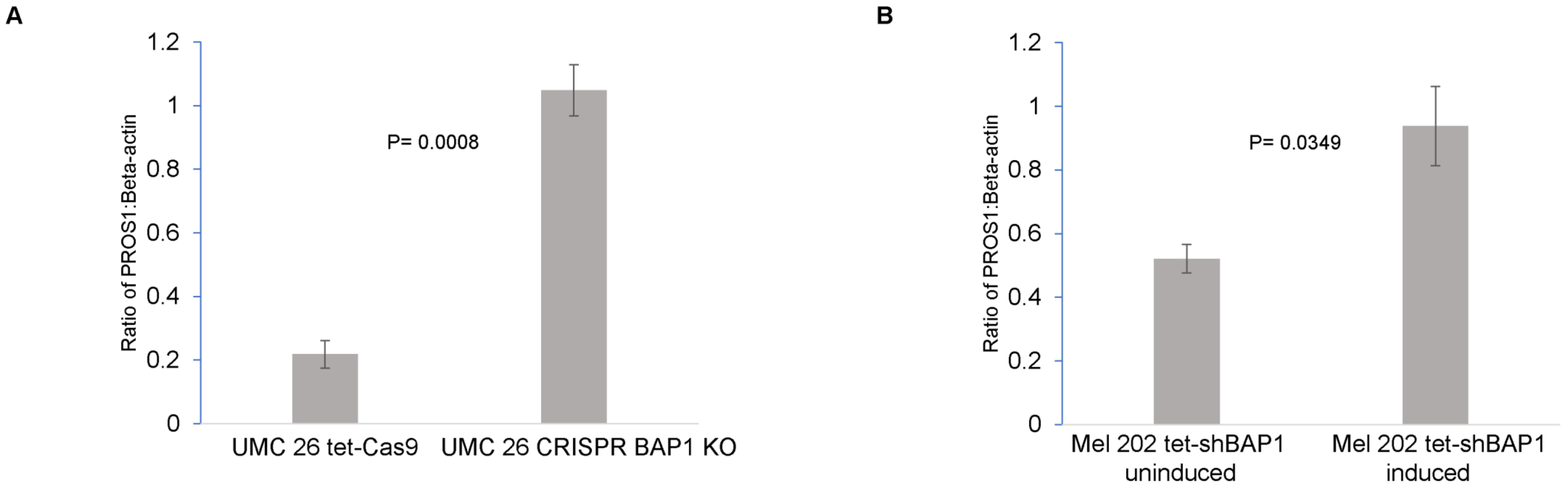

Figure S1. (**A**) Western blots of lysates from UMC026 tet-Cas9 and UMC026 CRISPR BAP1-KO cells reveal increase of PROS1 protein following BAP1 KO by densitometric quantitation of multiple blots (n=3). (**B**) Western blots of lysates from Mel202 tet-shBAP1 uninduced and Mel202 tet-shBAP1 uninduced reveal increase of PROS1 protein following BAP1 knockdown by densitometric quantitation of multiple blots (n=3). Error bars=SEM.

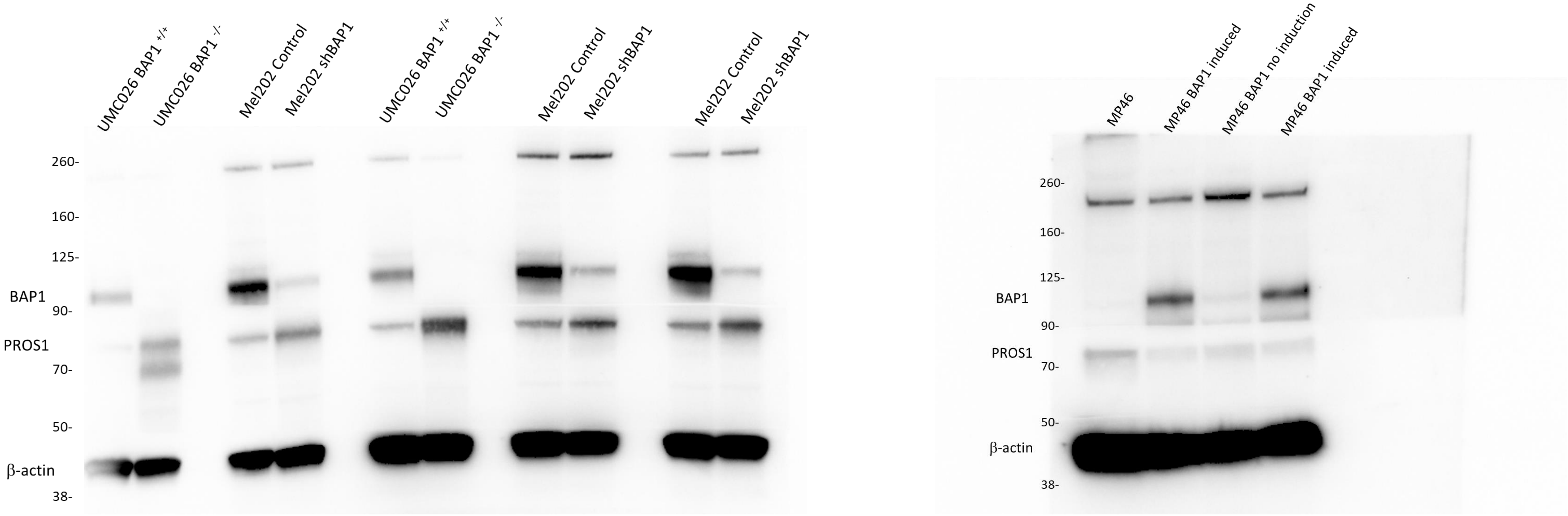

Figure S2. Full Western blot images for Figure 1

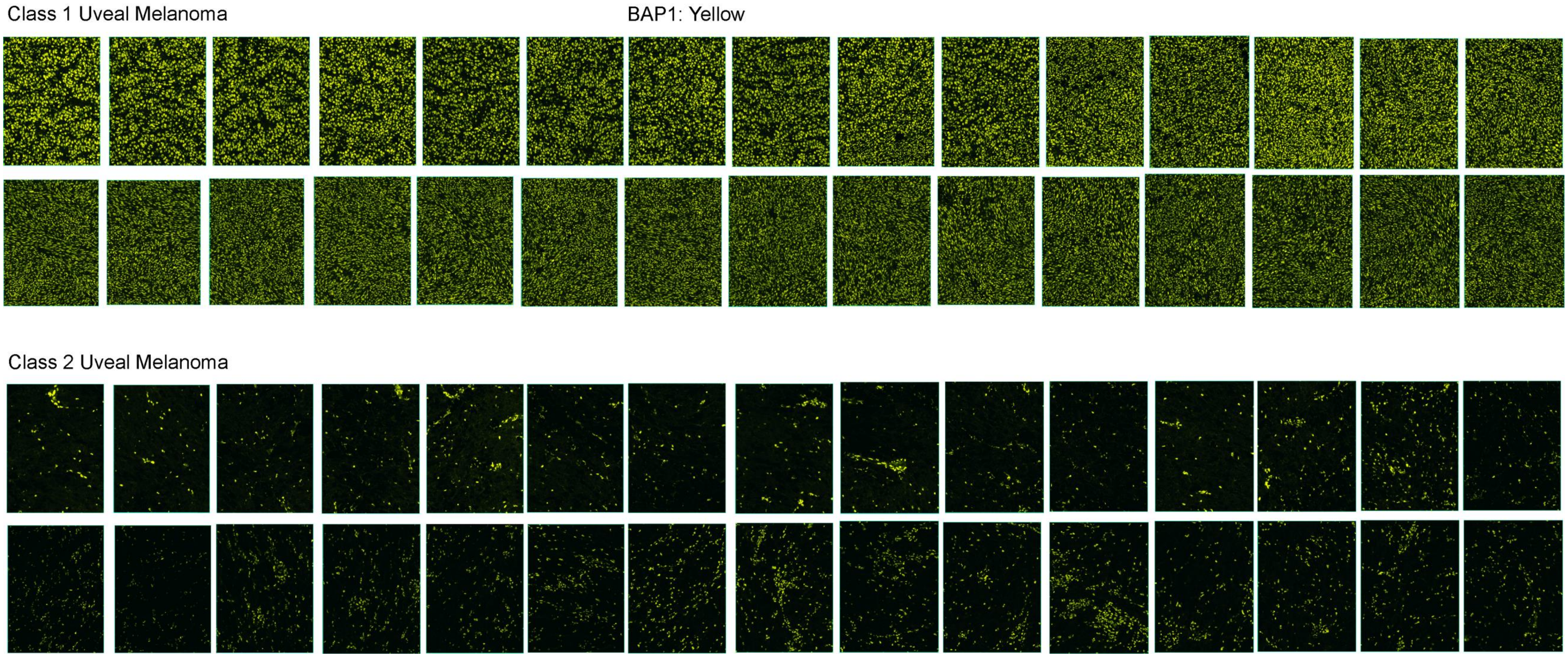

Figure S3. Immunohistochemistry for BAP1 from 60 regions from class 1 (UMM65, UMM79) and class 2 (UMM63, UMM69) uveal melanomas.

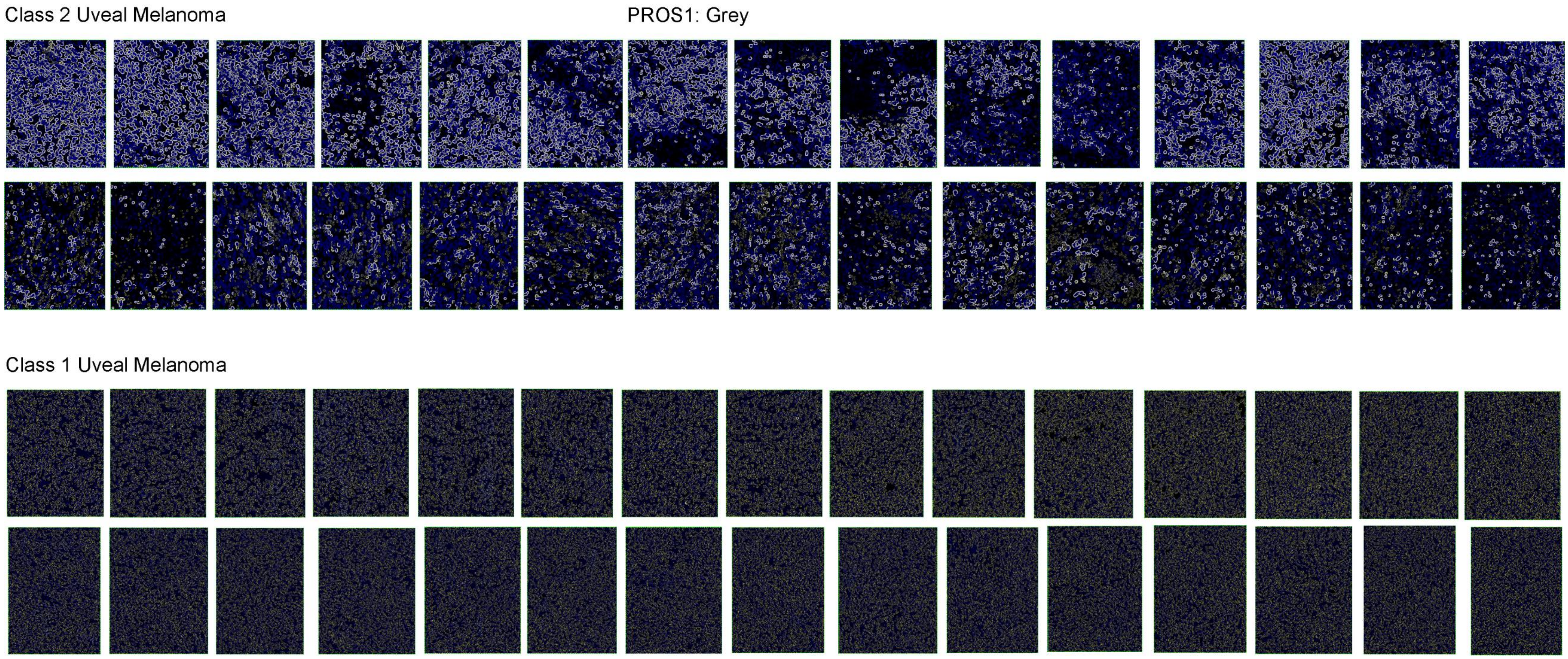

Figure S4. Immunohistochemistry for PROS1 from 60 regions from class 1 (UMM65, UMM79) and class 2 (UMM63, UMM69) uveal melanomas.

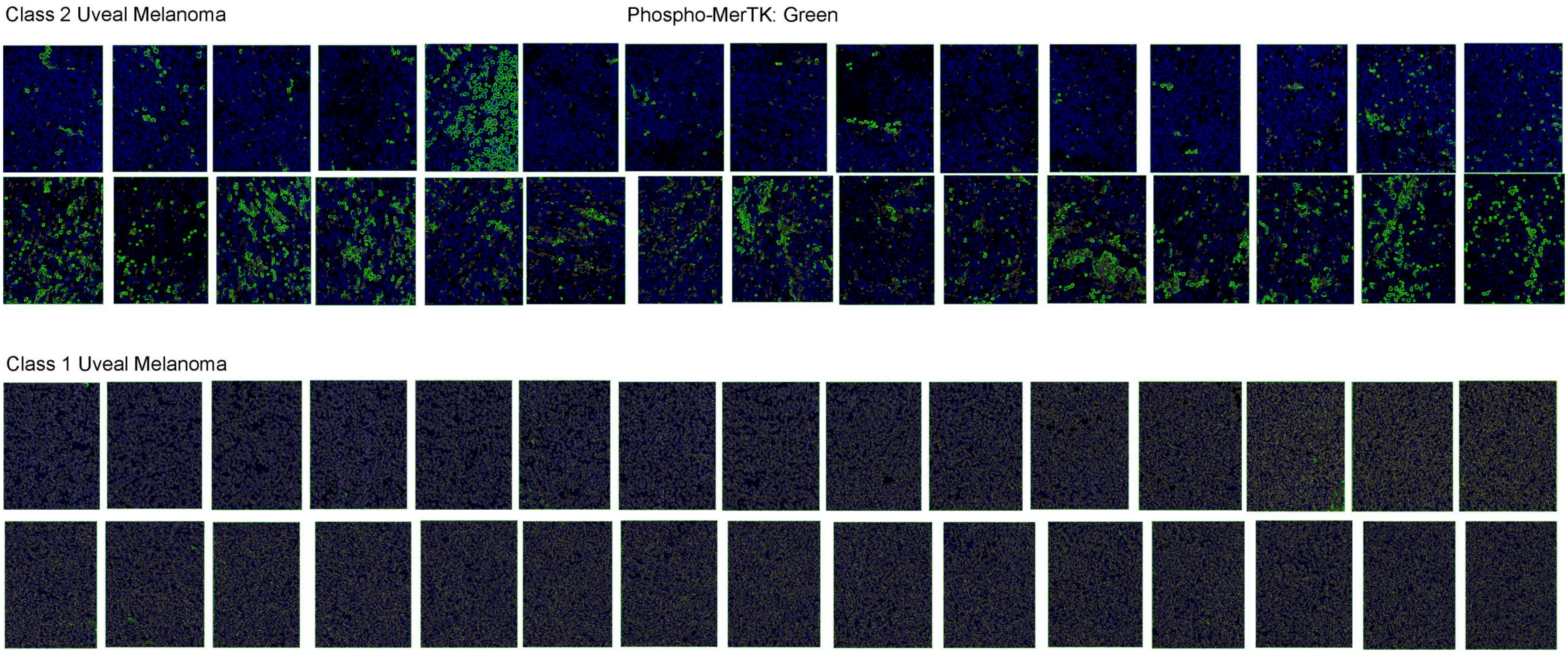

Figure S5. Immunohistochemistry for phospho-MERTK regions from class 1 (UMM65, UMM79) and class 2 (UMM63, UMM69) uveal melanomas.

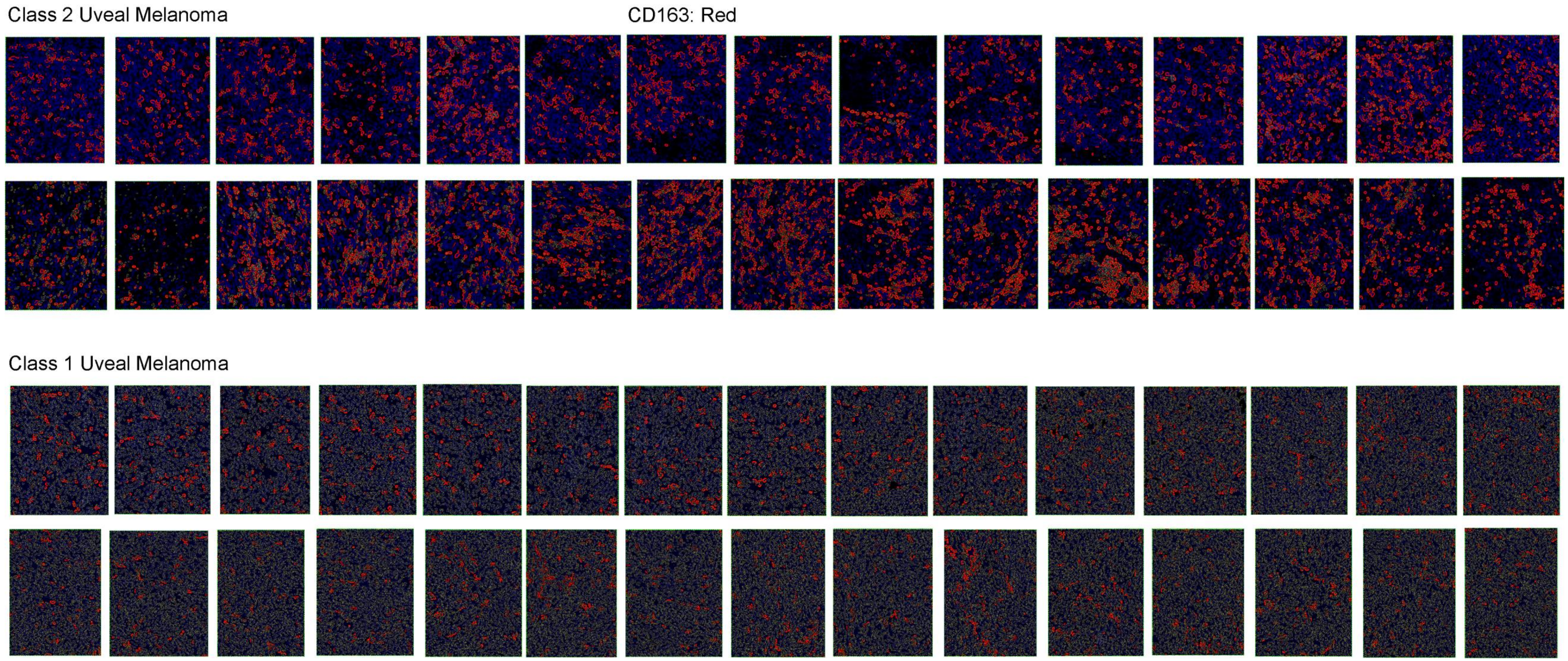

Figure S6. Immunohistochemistry for CD 163 from 60 regions from class 1 (UMM65, UMM79) and class 2 (UMM63, UMM69) uveal melanomas.

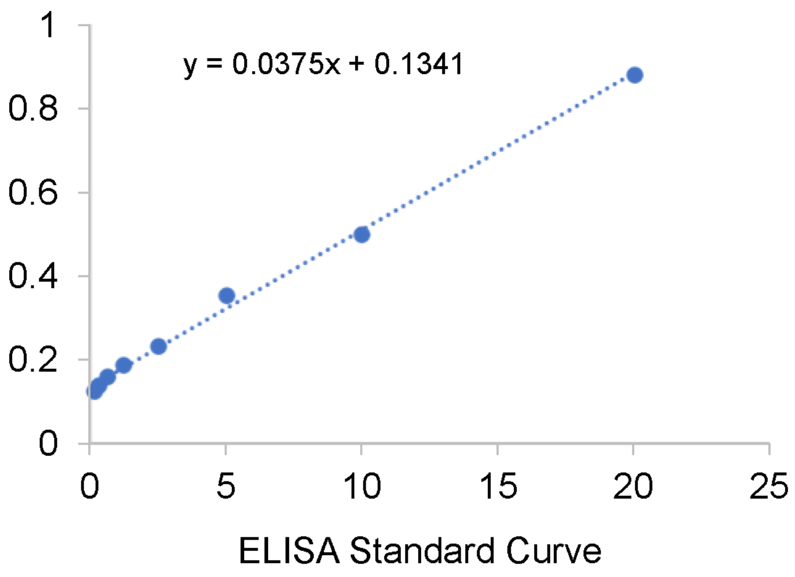

Figure S7. ELISA standard curve documenting linearity of analyte concentration.

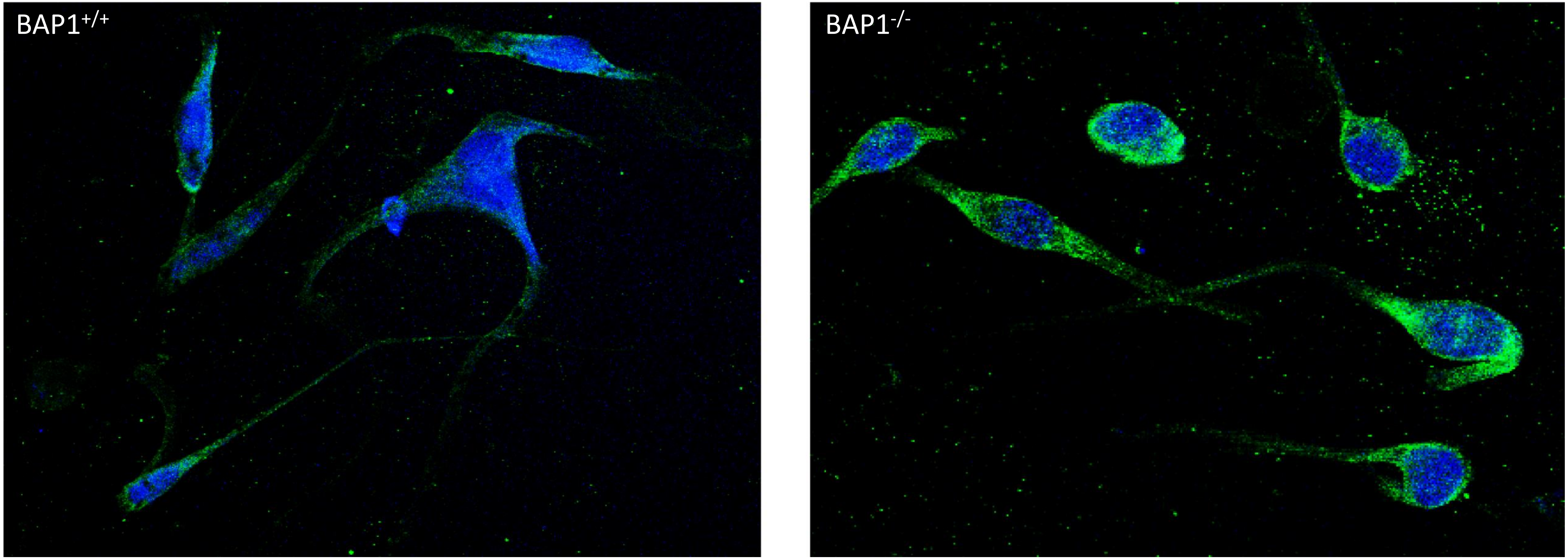

Figure S8. Confocal microscopy images of UMC026 cells with or without BAP1 knockout immunostained for PROS1 (green). Blue= DAPI nuclear stain.

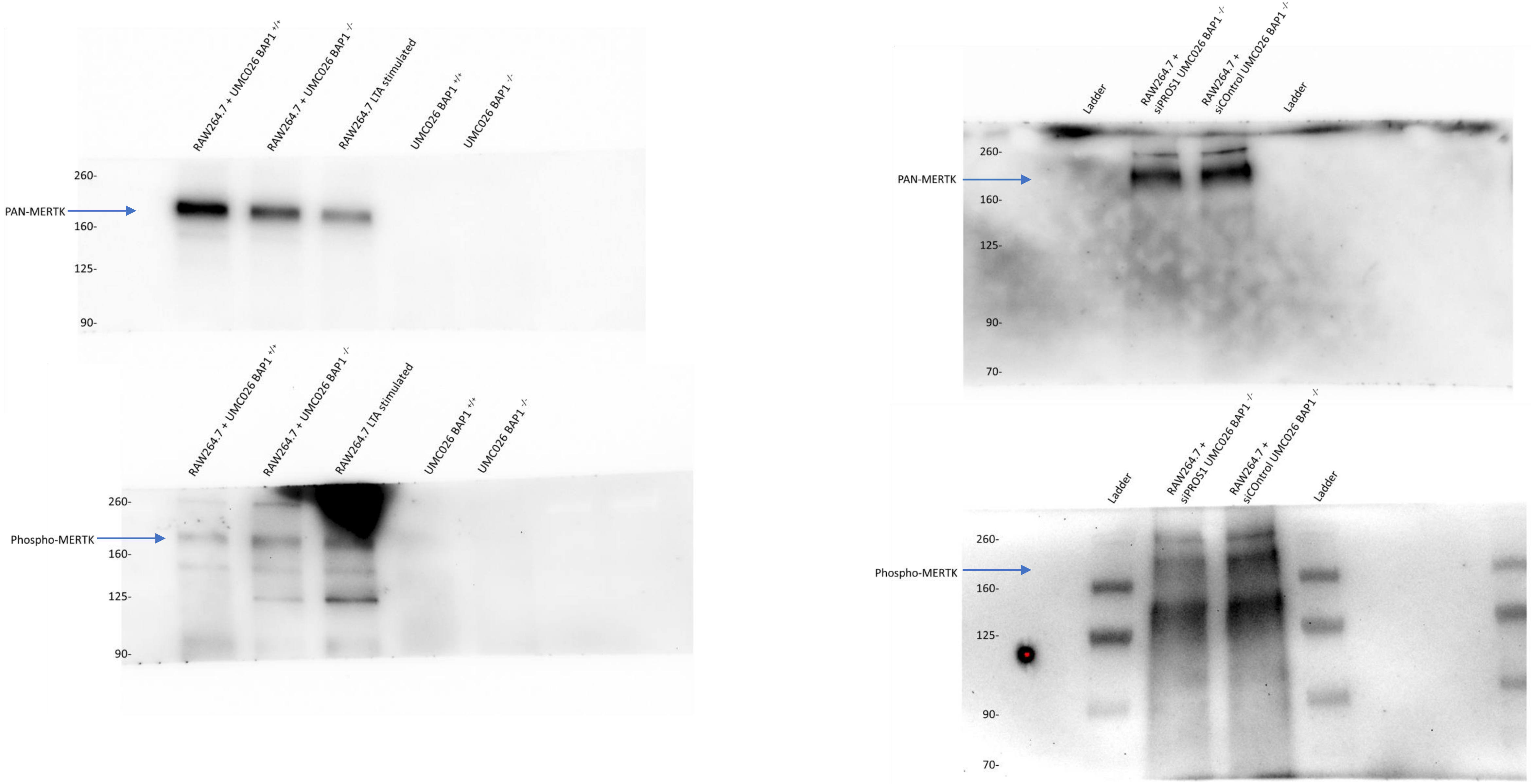

Figure S9. Full Western blot images for Figure 4.

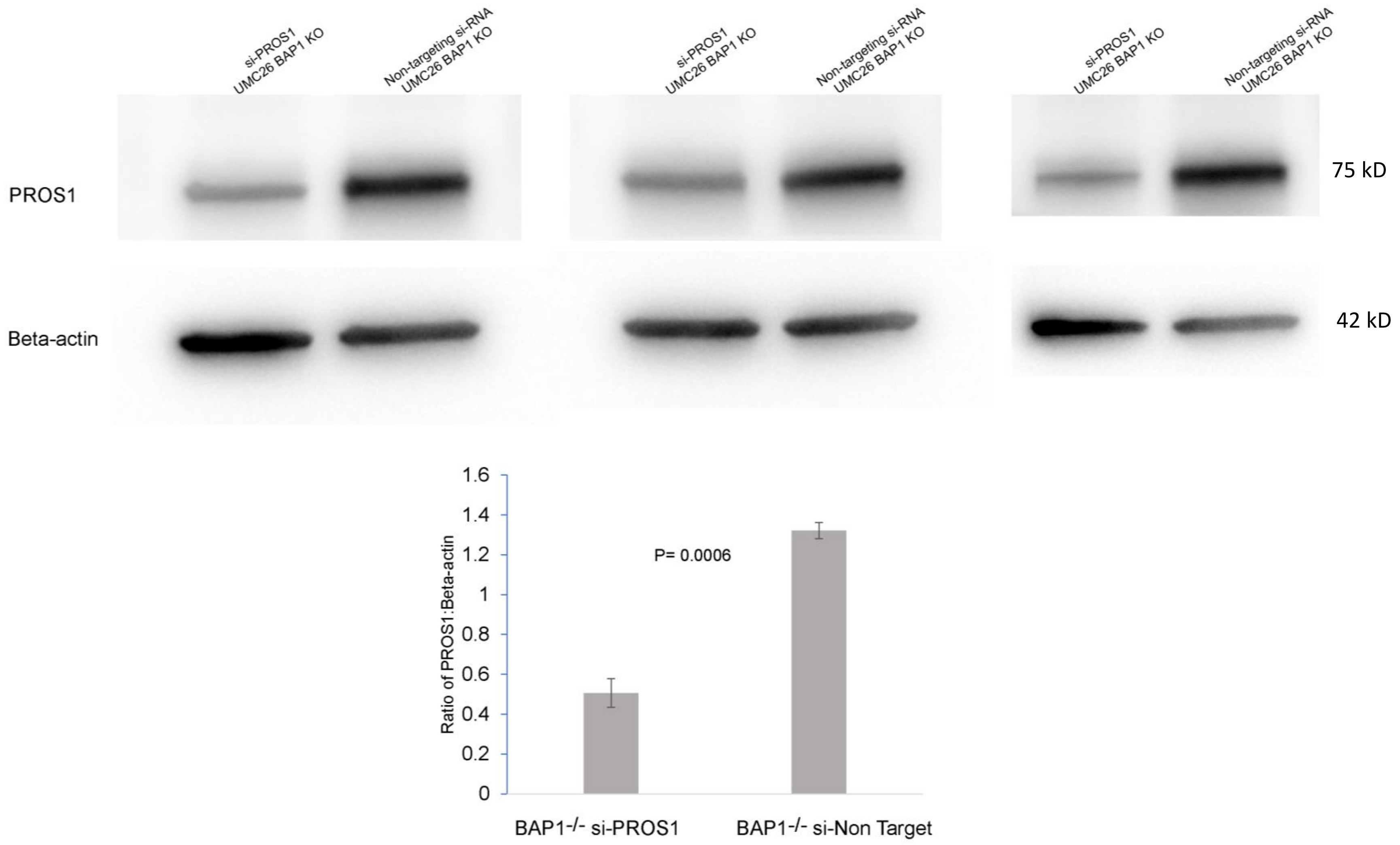

Figure S10. Confirmation of PROS1 knockdown in UMC026 BAP1-negative cells used in RAW cell co-culture functional assays. Western blots of lysates from the UMC026 BAP1-KO cells used in co-culture experiments (**Figure 4E, F**) indicate siRNA knockdown of PROS1 in a range of 52-75% by densitometric quantitation of multiple blots (n=3). Error bars=SEM.

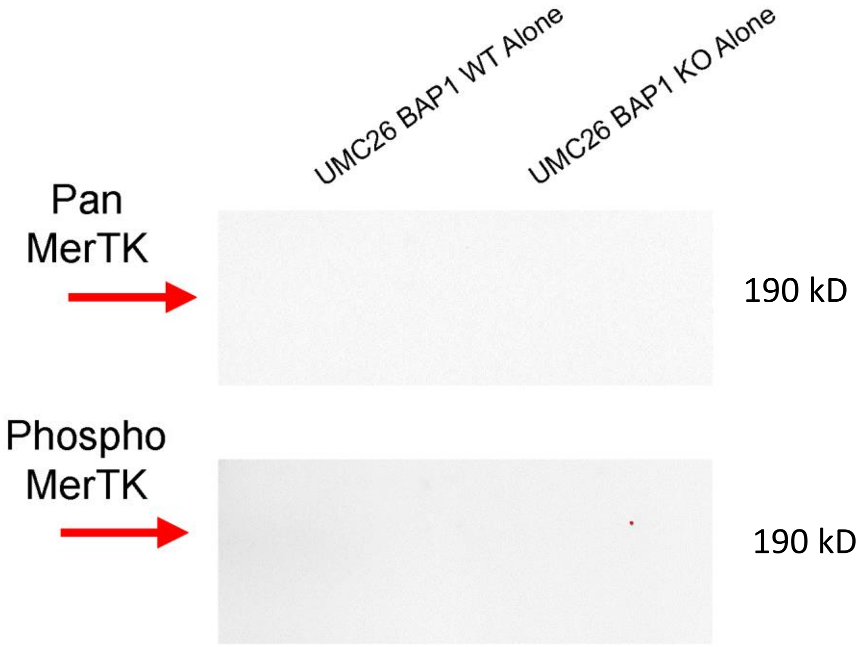

Figure S11. Immunoprecipitated MERTK expression is undetected when the melanocyte cell lines are cultured independently of RAW 264.7 cells.
